# Clockophagy in Alzheimer’s Disease: An Emerging Hypothesis Linking Circadian Dysfunction to Ferroptotic Vulnerability

**DOI:** 10.3390/cells15191721

**Published:** 2026-09-22

**Authors:** Jianqin Mao, Yudan Zhang, Ping Wang, Dongdong Qin

**Affiliations:** 1 The First Clinical Medical College, Yunnan University of Chinese Medicine, Kunming 650500, China; mm_0701@foxmail.com; 2 Health Science Center, Hubei Minzu University, Enshi 445000, China; zyd19910524@163.com; 3 Engineering Research Center, Hubei University of Chinese Medicine, Wuhan 430065, China; 4 Hubei Shizhen Laboratory, Wuhan 430065, China

**Keywords:** Alzheimer’s disease, sleep disorders, circadian rhythm, autophagy, ferroptosis, lipid peroxidation, clockophagy

## Abstract

Alzheimer’s disease (AD) is frequently associated with sleep disturbances and circadian dysfunction, which may precede or accompany clinical manifestations and have been associated with amyloid-β (Aβ) pathology, tau abnormalities, oxidative stress, lipid peroxidation, and neuronal vulnerability. Increasing evidence suggests that circadian regulation intersects with multiple cellular processes implicated in AD pathogenesis, including autophagy and ferroptosis. Among these mechanisms, clockophagy, an SQSTM1/p62-mediated selective autophagic process involved in BMAL1 degradation, has recently emerged as a potential molecular link between circadian regulation and cellular stress responses. In this review, we summarize current evidence connecting sleep disruption, circadian dysfunction, lipid peroxidation, and ferroptotic susceptibility in AD, while highlighting the current limitations of mechanistic evidence. We further discuss the molecular basis of clockophagy and its potential relationship with BMAL1 regulation, oxidative lipid damage, and ferroptosis. Collectively, clockophagy is currently viewed as an emerging hypothesis that may contribute to AD-related pathological processes. Finally, we discuss potential therapeutic implications, including approaches targeting sleep and circadian organization, BMAL1-associated regulatory pathways, and lipid oxidative stress. Further studies are required to determine whether modulation of clockophagy can influence AD-related neurodegenerative processes.

## 1. Background

Since its first clinicopathological description by Alois Alzheimer in 1906, Alzheimer’s disease (AD) has been recognized as the most common cause of dementia and is neuropathologically characterized by extracellular amyloid-β (Aβ) plaques and intracellular neurofibrillary tangles composed of hyperphosphorylated tau [1]. These pathological hallmarks contribute to synaptic dysfunction, neuronal loss, and progressive cognitive decline. According to global epidemiological estimates, dementia affected approximately 57.4 million individuals worldwide in 2019, with AD accounting for approximately 60–70% of dementia cases [2]. The prevalence and socioeconomic burden of AD are expected to increase substantially with population aging [3]. Beyond age and genetic susceptibility, modifiable factors, including vascular health, physical activity, social engagement, and sleep disorders, may influence AD risk and progression [4]. Among these factors, sleep disturbances have received increasing attention because of their high prevalence, clinical accessibility, and potential for intervention.

Increasing evidence suggests that sleep disturbances and circadian abnormalities are not merely clinical manifestations of AD but may also participate in disease-related biological processes [5]. These alterations include fragmented sleep, altered sleep timing, reduced circadian amplitude, impaired melatonin rhythms, and disrupted rest–activity cycles [6]. Although sleep regulation and circadian timing are closely interconnected, they represent distinct biological processes that may influence AD through partially overlapping but not identical mechanisms [7]. Various sleep-related and circadian perturbations, including sleep deprivation, obstructive sleep apnea (OSA), circadian misalignment, and alterations in core clock components, have been associated with oxidative stress, impaired proteostasis, neuroinflammation, and altered cellular homeostasis [8].

Circadian rhythm dysfunction in AD is characterized by attenuated and fragmented rest–activity rhythms, delayed melatonin secretion, and phase-delayed and/or attenuated core body temperature rhythms. These disturbances are often accompanied by altered sleep architecture, including disrupted NREM–REM sleep cycling [9,10,11]. The circadian system is primarily organized around the suprachiasmatic nucleus (SCN), which coordinates the synchronization of peripheral clocks. Recent studies suggest that sleep and circadian abnormalities may occur before overt clinical manifestations and are associated with Aβ accumulation, tau pathology, impaired glymphatic clearance, neuroinflammation, and cognitive decline [12,13,14]. However, whether these disturbances actively contribute to AD-related biological changes or primarily reflect underlying disease processes remains an open question.

Given these connections, exploring the molecular mechanisms linking sleep and circadian disturbances to AD progression is of considerable scientific and clinical interest. Existing research has begun to outline this relationship, but a unified framework integrating behavioral phenotypes with molecular pathways remains lacking. AD pathogenesis involves complex interactions among autophagic dysfunction, oxidative stress, neuroinflammation, lipid peroxidation, and ferroptotic vulnerability, many of which are associated with circadian regulation [15,16,17].

Within this context, clockophagy, a selective autophagic process in which SQSTM1/p62 mediates the degradation of BMAL1, has emerged as a potential mechanism connecting circadian regulation with lipid peroxidation and ferroptosis, although its role in AD remains largely inferential and requires further experimental validation [18]. In this review, we summarize current evidence linking sleep disturbance and circadian dysfunction to AD pathology, focusing on their relationships with autophagic regulation and lipid peroxidation associated ferroptotic vulnerability. We further examine whether clockophagy may represent a potential molecular interface connecting sleep–circadian disruption, BMAL1 regulation, and ferroptotic susceptibility in AD.

## 2. Literature Search Strategy

A structured literature search was conducted using PubMed, Google Scholar, and Scopus, with the search completed on 30 June 2026. Search terms included three categories: disease-related terms (“Alzheimer’s disease,” “mild cognitive impairment,” and “cognitive decline”), sleep/circadian-related terms (“sleep,” “sleep disorders,” “circadian rhythm,” and “obstructive sleep apnea”), and mechanism-related terms (“BMAL1,” “autophagy,” “clockophagy,” “ferroptosis,” and “lipid peroxidation”). Boolean operators were used to combine related concepts. Peer-reviewed English-language clinical studies, animal studies, and in vitro studies were considered, with recent publications prioritized while retaining seminal studies where appropriate. Primary studies were preferentially cited for specific mechanistic findings. As this is a narrative review, evidence was synthesized qualitatively, with particular attention to distinguishing direct evidence from AD-related models and mechanistic evidence derived from non-AD systems. For clarity, evidence is considered throughout this review at three levels: AD-specific evidence, mechanistic evidence derived from non-AD experimental models, and biologically plausible but currently hypothetical links. This evidence hierarchy is summarized in Table 1.

## 3. Pathogenesis of Alzheimer’s Disease

### 3.1. Aβ and Tau Pathology and Neuronal Vulnerability

AD is neuropathologically characterized by extracellular Aβ deposits, intracellular neurofibrillary tangles composed of hyperphosphorylated tau, synaptic dysfunction, and progressive neuronal loss [33]. Aβ is generated through sequential cleavage of amyloid precursor protein by β- and γ-secretases, with Aβ42 displaying a particularly high propensity for aggregation [34,35]. Tau normally contributes to microtubule stability and axonal transport, whereas pathological hyperphosphorylation, misfolding, and aggregation impair cytoskeletal organization and neuronal function [36,37]. Aβ and tau pathology, however, does not occur in isolation. Mitochondrial dysfunction, neuroinflammation, oxidative stress, impaired proteostasis, altered autophagy–lysosome function, lipid metabolic abnormalities, and regulated cell-death pathways interact with these canonical pathological features [38,39,40,41]. Among these processes, lipid peroxidation is particularly relevant because neuronal membranes are enriched in oxidation-sensitive polyunsaturated fatty acids (PUFAs), and oxidative lipid damage can perturb membrane integrity, organelle function, protein homeostasis, and cellular signaling [42,43,44]. Rather than representing a single unifying cause of AD, lipid peroxidation may therefore act as a potential convergent mediator of AD-related cellular stress, linking several pathological processes associated with neuronal vulnerability.

### 3.2. Lipid Peroxidation as a Convergent Hub in AD Pathogenesis

Lipid peroxidation is an oxidative process that primarily affects membrane-associated polyunsaturated fatty acids (PUFAs), including arachidonic acid and adrenic acid. Once initiated, lipid peroxidation can propagate through radical chain reactions, generating lipid hydroperoxides and reactive aldehydes such as malondialdehyde (MDA) and 4-hydroxynonenal (4-HNE) [45,46]. These products can modify membrane lipids, proteins, and nucleic acids, thereby altering membrane properties, enzyme activity, organelle function, and intracellular redox homeostasis [47]. The brain is particularly susceptible to lipid oxidative damage because of its high oxygen consumption, abundant lipid content, enrichment in oxidation-sensitive PUFAs, and substantial metabolic demand [48]. In neuronal and glial cells, excessive lipid peroxidation can compromise membrane integrity and signaling, impair organelle function, and amplify oxidative stress [49,50,51]. Increased lipid oxidative damage has been reported in AD. Elevated levels of 4-HNE, MDA, and other lipid peroxidation products have been detected in AD brain tissue and biological fluids [52,53], while experimental AD models have also shown increased lipid peroxidation, including elevated 4-HNE and MDA in APP/PS1 mice [54,55]. In a non-AD neuronal model, Van Acker et al. further demonstrated the relevance of lipid-redox regulation to ferroptotic vulnerability. Neuroglobin overexpression protected SH-SY5Y neuronal cells against erastin-induced ferroptosis, with associated changes in the iron-handling protein PCBP3, the ferroptosis-related stress protein HSPB1, Nrf2 upregulation, and reduced MDA accumulation [56]. Collectively, these findings support a role for lipid peroxidation in AD-associated cellular stress and neuronal vulnerability, although its precise contribution to disease progression remains to be established.

### 3.3. Lipid Peroxidation in Aβ and Tau Pathology

Aβ and tau pathology are closely associated with lipid oxidative stress in AD. Aβ species, particularly oligomeric Aβ, can interact with neuronal membranes and redox-active metals such as Fe^2+^ and Cu^2+^, promoting ROS generation and lipid peroxidation [57,58]. The resulting products, including MDA and 4-HNE, can impair membrane integrity, protein function, and cellular signaling, thereby increasing neuronal vulnerability [59,60,61]. Experimental studies have demonstrated that Aβ40 and Aβ42 induce protein oxidation, lipid peroxidation, and neuronal injury, while Met35-associated radical formation contributes to lipid peroxidation chain reactions [62,63]. Aβ oligomers can also increase lipid-droplet accumulation and oxidative stress in astrocytes [64]. Tau is likewise involved in lipid-redox homeostasis, with experimental evidence showing its requirement for glial lipid-droplet responses that protect against neuron-derived oxidative stress [65,66]. Conversely, arachidonic acid and oxidized lipids promote tau aggregation, whereas 4-HNE forms adducts with phosphorylated tau and enhances its aggregation in experimental systems [67]. Of particular significance is the reactive aldehyde 4-HNE that is upregulated in the AD brain. 4-HNE can create adducts with phosphorylated tau and initiate tau aggregation in both in vitro and cellular studies [68,69]. Lipid peroxidation products can additionally impair lysosomal and proteostatic function and promote the formation of protein aggregates that may facilitate tau fibrillization [70,71]. Collectively, these findings demonstrate reciprocal interactions between Aβ/tau pathology and lipid peroxidation across experimental systems, supporting lipid oxidative stress as an important component of AD-related pathology. Figure 1 depicts the proposed influence of lipid peroxidation processes in AD.

## 4. Circadian Rhythms and Sleep in Alzheimer’s Disease

### 4.1. The Circadian Clock in the Brain

Circadian rhythms are endogenous biological oscillations with an approximately 24 h periodicity that coordinate diverse physiological processes, including sleep–wake cycles, metabolism, endocrine regulation, immune function, and cognition [72,73,74]. At the molecular level, mammalian circadian rhythms are generated by interlocking transcription–translation feedback loops (TTFLs). In the core loop, the CLOCK–BMAL1 heterodimer activates transcription of Period (*Per1/2*) and Cryptochrome (*Cry1/2*) genes through E-box elements. Accumulated PER and CRY proteins subsequently form repressive complexes that inhibit CLOCK–BMAL1-dependent transcription, thereby generating an approximately 24 h oscillation [75,76]. Additional regulatory loops involving REV-ERBα/β and RORα/β modulate BMAL1 expression and contribute to the stability and precision of circadian oscillations [77].

At the systems level, the suprachiasmatic nucleus (SCN) serves as the central pacemaker and coordinates rhythmic outputs to other brain regions and peripheral tissues [78,79]. SCN-related pathways involving the paraventricular nucleus (PVN), preoptic area, dorsomedial hypothalamus (DMH), subparaventricular zone (SPVZ), and locus coeruleus (LC) contribute to the temporal regulation of endocrine function, sleep–wake behavior, feeding, autonomic activity, arousal, stress responses, and cognition [80,81,82,83,84]. Circadian signals also modulate hippocampal synaptic plasticity and memory-related processes [85,86]. Circadian timing is synchronized by external cues, or Zeitgebers. Light is the dominant Zeitgeber for the SCN, with photic information conveyed primarily by intrinsically photosensitive retinal ganglion cells (ipRGCs) through the retinohypothalamic tract [87], whereas feeding, activity, temperature, and social timing can additionally influence peripheral or behavioral rhythms [88,89]. Disruption of this temporal organization may manifest as altered phase, reduced amplitude, impaired entrainment, or desynchronization between central and peripheral clocks [90,91,92]. Although sleep and circadian regulation are tightly coupled, disturbances in sleep homeostasis and circadian timing may exert partly distinct effects on neural and metabolic function while converging on several pathways relevant to AD pathophysiology.

### 4.2. Circadian Dysregulation in Alzheimer’s Disease

Circadian abnormalities are commonly observed in AD and may include fragmented rest–activity rhythms, reduced circadian amplitude, altered sleep–wake timing, and impaired synchronization of behavioral and physiological rhythms [93,94]. Circadian dysfunction in AD may involve alterations in SCN output, molecular clock regulation, environmental entrainment, and coordination among central and local oscillators [95].

The SCN is particularly relevant to circadian dysfunction in AD because alterations in its neuronal and molecular organization may weaken the generation and coordination of circadian signals [96]. Neuropeptidergic changes within the SCN have also been reported in human AD. A postmortem study identified a significant reduction in VIP-expressing SCN neurons in female patients with presenile AD, although this effect was influenced by age and sex [97]. In a separate human postmortem study, AVP mRNA expression in the SCN was markedly reduced in AD compared with age- and time-of-death-matched controls, accompanied by a loss of the normal day–night variation in AVP gene expression [98]. Together, these findings suggest that alterations in SCN neuropeptidergic signaling may contribute to the weakening of circadian rhythmicity observed in AD.

Circadian alterations in AD are not restricted to the SCN. Core clock components, including *CLOCK*, *BMAL1*, *PER*, and *CRY* proteins, are expressed in multiple neural cell types and brain regions, where they contribute to local circadian oscillations [99,100]. Alterations in central pacemaker function together with changes in local clock regulation may therefore contribute to disrupted temporal organization in AD. In addition, reduced MT1 melatonin receptor expression in the SCN has been reported in aging and AD [101], providing evidence of altered melatonin-related circadian signaling.

Aging and AD are associated with alterations in circadian organization, while environmental factors such as reduced or irregular light exposure may further influence circadian entrainment. Age-related structural and functional changes in the retina have also been described. In addition, retinal biomarkers, including retinal gliosis and retinal nerve fiber layer alterations, have been associated with biomarkers of preclinical AD [102,103,104]. However, direct evidence that these retinal abnormalities impair photic entrainment of the SCN in AD remains limited.

Overall, circadian disruption in AD appears to involve abnormalities across behavioral, neural, molecular, and environmental levels. Nevertheless, whether such disruption acts as a causal contributor, a consequence of neurodegeneration, or both is likely to depend on disease stage and biological context.

### 4.3. Sleep Disturbance and Alzheimer’s Disease

Sleep is regulated by interactions between circadian timing and homeostatic sleep pressure. Whereas the circadian system organizes the timing of sleep and wakefulness, homeostatic sleep pressure increases during wakefulness and declines during sleep [105]. Sleep architecture includes non-rapid eye movement (NREM) and rapid eye movement (REM) sleep, which contribute differently to synaptic homeostasis, memory consolidation, and cognitive processing [106,107]. Sleep deprivation can impair cognitive functions associated with the prefrontal cortex, including attention, executive function, and short-term memory [108]. In patients with mild cognitive impairment or AD, sleep disturbances are common and include insomnia, sleep fragmentation, reduced slow-wave sleep, reduced REM sleep, excessive daytime sleepiness, and sleep-disordered breathing [109]. These alterations are particularly relevant to cognition because slow-wave activity and sleep spindles during NREM sleep, together with REM sleep, participate in memory processing and the consolidation of hippocampal-dependent information [110].

Environmental timing cues also influence sleep–wake organization through circadian entrainment. Light is the dominant Zeitgeber for the central circadian system [111]. In AD, reduced daytime light exposure and inappropriate nocturnal light exposure may weaken circadian entrainment and destabilize sleep–wake rhythms [112]. Timed bright-light exposure has therefore been investigated as a non-pharmacological intervention, although clinical findings remain heterogeneous because of differences in light intensity, spectrum, timing, treatment duration, and study design [113].

Sleep may also interact with AD-related pathology through brain clearance and metabolic pathways. Experimental evidence, particularly from animal models, suggests that slow-wave sleep facilitates cerebrospinal fluid (CSF) movement and may enhance clearance of soluble metabolites, including Aβ and tau-related species [114,115]. Rest–activity fragmentation has also been associated with greater Aβ burden and cognitive decline in older adults [116,117]. However, the contribution of glymphatic clearance to human AD remains incompletely established. In *Drosophila*, sleep has additionally been shown to promote removal of oxidized brain lipids accumulated during wakefulness [118], although whether an analogous mechanism exists in mammals or contributes to AD remains unknown.

Sleep–wake disturbances may further interact with tau pathology. Tau accumulation in arousal-related brain regions has been described during early AD-related neurodegeneration [119]. Acute sleep loss has been associated with increased CSF tau levels and altered CSF-to-blood clearance, suggesting that sleep–wake disturbances may influence tau-related processes [120]. In addition, enhancing sleep through orexin receptor blockade has been shown to reduce hyperphosphorylated tau in animal models [121]. Together, these observations support a close relationship between sleep–wake regulation and tau-related pathology. In parallel with its effects on brain clearance and lipid homeostasis, sleep disruption may promote metabolic and oxidative stress, creating conditions that increase neuronal vulnerability and facilitate AD-related pathological processes. These converging relationships are summarized in Figure 2. A comparative overview of the major features and mechanistic relationships among AD, sleep disturbance, circadian disruption, autophagy, ferroptosis, and clockophagy is provided in Table 2.

## 5. The Potential Role of Clockophagy in the Connection Between Sleep and AD

### 5.1. BMAL1-Centered Autophagic Regulation

BMAL1 is a core transcriptional regulator of the circadian clock, but its functional relevance extends beyond rhythm generation. As discussed above, clockophagy provides a selective autophagy-based mechanism through which BMAL1 protein stability may be linked to ferroptotic signaling [18,122]. This section therefore focuses on the autophagic regulation of BMAL1 and the reciprocal influence of BMAL1 on autophagic processes in neural cells.

The stability of BMAL1 is controlled by coordinated proteasomal and autophagic degradation pathways. In this process, the autophagy receptor SQSTM1/p62 links ubiquitin signaling to lysosomal clearance by recognizing ubiquitinated BMAL1 and directing it toward selective autophagic degradation. SUMOylation of BMAL1 at K259 contributes to the regulation of BMAL1 stability and circadian transcriptional activity [123]. SQSTM1/p62 can recognize ubiquitinated BMAL1 through its ubiquitin-binding domain and promote its autophagic degradation, functionally, CLOCK-induced degradation of Flag BMAL1 is impaired in cells lacking p62, supporting the role of SQSTM1/p62 in BMAL1 autophagic degradation [124]. In addition, p62 deficiency disrupts BMAL1 protein homeostasis, increases the expression of BMAL1 target genes, and reduces the transcript levels of *Bmal1* and *Clock*, suggesting that p62-mediated regulation of BMAL1 protein stability may contribute to the fine tuning of circadian clock function [125].

BMAL1 is not only a substrate of autophagic degradation but also participates in the regulation of autophagy in neural cells. In astrocytes, genetic *Bmal1* deficiency has been associated with increased endocytosis, enhanced lysosome-dependent protein cleavage, and increased endolysosomal activity [126]. In hippocampal neurons, BMAL1 upregulates STX17 expression and promotes autophagosome–lysosome fusion in experimental AD models [32]. These observations indicate that BMAL1 contributes to the regulation of autophagic processes in neural cells, with effects that may differ among cell types.

Based on these observations, BMAL1 is not only subjected to autophagic degradation but also participates in the regulation of autophagic and lysosome-related processes in neural cells. SQSTM1/p62-mediated regulation of BMAL1 protein stability may contribute to circadian protein homeostasis, whereas BMAL1 dependent modulation of autophagic processes may influence cellular clearance in neural cells. Together, these reciprocal interactions between circadian regulation and autophagy provide a potential mechanistic framework through which circadian disruption may contribute to proteostatic imbalance in neurodegenerative diseases. These reciprocal interactions between BMAL1 and autophagic regulation are summarized in Figure 3.

### 5.2. Autophagy, Ferroptosis, and Lipid Peroxidation in AD

In the area of AD pathology, autophagy seems to alter through various phases, acting as a protective mechanism of clearance under either physiological or early compensatory conditions, but possibly favoring development of the disease when disruption of autophagic flow and lysosomal degradation starts to occur. Basal autophagy has an important role in clearance of toxic peptides involved in Aβ pathology and hyperphosphorylated tau proteins [127]. LC3-associated endocytosis promotes clearance of Aβ and inhibition of neurodegenerative processes in AD mice [128]. In contrast, the lack of important autophagy genes such as ATG5 and ATG7 compromises the functioning of autophagy, hence destabilizing synaptic protein integrity and cognitive abilities [129,130].

Glial cell studies show that autophagy plays a part in clearing AD-related proteins. Studies of Aβ-exposed human astrocytes and mouse models of AD suggest that astrocytic autophagy has plastic changes allowing modulation of Aβ clearance and cognitive performance. The blockade of LC3B or SQSTM1 in astrocytes disrupts this clearance, whereas increased expression of astrocytic LC3B relieves the pathology of AD [131]. AD pathology progresses by changing autophagy function from protective clearance to dysfunctional state. In such a new state, autophagy is marked by the presence of autophagosomes, reduced autophagic activities, and lysosomal degradation capabilities. Consequently, there will be inability to eliminate Aβ and tau. As a result, accumulation of these two leads to further burdening the clearance mechanism, resulting in a cycle of toxic proteins deposition and failure of autophagy [132].

The connection between autophagy and lipid peroxidation adds to the complexity of AD pathology. Lipid peroxidation products can serve as signals of stress in autophagic responses. For instance, ROS-dependent lipid peroxidation correlates with autophagic activation [133]. The lipid peroxidation product 4-HNE can trigger autophagy through several pathways. It may promote the release of BECN1 by activating endoplasmic reticulum stress pathways such as PERK and JNK signaling, while increased HMOX1 expression can enhance the conversion of LC3-I to LC3-II [134,135]. MDA can also bring about the production of proteins related to autophagy such as LC3 and ATG7 [136]. However, excessive lipid peroxidation might also impair the function of autophagy. 4-HNE can interfere with mitochondrial function and autophagic flux, leading to reduced elimination of aberrant proteins, including Aβ and tau, from neurons [137]. Lipid peroxidation products also exert negative effects on lysosomes by inhibiting their enzymes and consequently impairing autophagic degradation through the development of lipofuscin [138]. However, although 4-HNE can increase LC3-II levels, lysosomal dysfunction may block normal autophagic flux, resulting in impaired clearance and ultimately contributing to neuronal cell death [139]. This indicates that the interplay between lipid peroxidation and autophagy might contribute to proteostatic failure in AD.

Ferroptosis is an iron-dependent form of regulated cell death characterized by the excessive accumulation of phospholipid peroxides under conditions of insufficient lipid peroxide detoxification [140]. First described in 2012, ferroptosis is closely associated with the SLC7A11–GSH–GPX4 axis. Erastin suppresses SLC7A11 activity, thereby inhibiting cystine uptake and reducing glutathione availability, whereas RSL3 directly inhibits GPX4 and impairs the enzymatic detoxification of lipid hydroperoxides [141].

Iron promotes lipid ROS generation through Fenton chemistry and lipoxygenase-mediated lipid oxidation, thereby increasing oxidative damage to cellular membranes [142]. Recent evidence further shows that GPX4 dysfunction can directly drive ferroptotic neurodegeneration in mammalian and human neuronal models, with Alzheimer’s-like molecular signatures also reported [143]. Given the importance of lipid oxidative damage in AD-related neuronal vulnerability, ferroptosis may provide a mechanistic framework linking lipid-redox imbalance to regulated neuronal injury. However, it remains uncertain whether ferroptotic cell death represents a major driver of human AD progression. Importantly, increased ROS, MDA, 4-HNE, or lipid peroxidation alone does not establish ferroptosis. Identification of ferroptotic cell death should therefore be supported by more specific mechanistic evidence, such as iron dependence, disruption of the SLC7A11–GSH–GPX4 defense axis, accumulation of lipid hydroperoxides, and, where appropriate, rescue by ferroptosis-selective inhibitors.

Ferroptosis was originally recognized as distinct from apoptosis and autophagic cell death due to its morphological, biochemical, and genetic properties [144]. However, later investigations discovered that ferroptosis has a functional link with autophagy. Rather than being divided into strict autophagy-dependent and autophagy-independent forms, current evidence suggests that autophagic processes can modulate ferroptotic susceptibility in a context-dependent manner. In some experimental models, autophagy-related proteins such as ATG5, ATG7, and BECN1 contribute to ferroptotic responses [145,146], whereas ferroptosis can also occur without strict dependence on these autophagic processes [147]. Nonetheless, the role of autophagy in ferroptosis depends on the context under consideration. Indeed, while dysregulated autophagic degradation can promote ferroptosis, autophagy can also protect cells in some situations [122].

Different mechanisms related to autophagy have been identified in ferroptosis regulation, including selective autophagy, non-selective autophagy, and chaperone-mediated autophagy (CMA) [148]. Selective autophagy includes ferritinophagy, lipophagy, and clockophagy, which may modulate ferroptotic susceptibility through regulation of iron storage, lipid droplet degradation, and BMAL1 activity, respectively [18,146,149]. Non-selective autophagy may influence ferroptotic susceptibility through TMEM164/ATG5-dependent degradation of protective factors such as GPX4 and ferritin [150,151]. On top of that, STING-related autophagy is implicated in the degradation of GPX4 and initiation of ferroptosis [152]. CMA offers another pathway to GPX4 degradation: GPX4 has a KFERQ-like motif that HSC70 can recognize and transfer to the lysosomal receptor LAMP2A, thereby allowing lysosomal breakdown of GPX4 and reducing lipid peroxide detoxification activity [153,154]. The interaction between autophagy and ferroptotic regulation may help in elucidating the mechanisms behind proteostatic dysfunction, lipid redox imbalance, and regulated neuronal death in AD. The major autophagy-related pathways influencing ferroptotic susceptibility are summarized in Figure 4.

### 5.3. Mechanistic Evidence Linking Clockophagy to Ferroptosis

#### 5.3.1. The EGLN2/PHD1–HIF-1α Axis in Clockophagy-Induced Ferroptosis

In non-AD experimental models, clockophagy refers to the mechanism of selective autophagic degradation mediated by SQSTM1/p62 that is activated during the process of ferroptosis [18]. SQSTM1/p62 recognizes BMAL1/ARNTL and directs it toward lysosomal degradation. The pathway is activated generally by type II ferroptosis inducers such as RSL3 and FIN56 and not by type I inducers like erastin, sulfasalazine, and sorafenib [122]. BMAL1/ARNTL degradation takes place with the core autophagy proteins, ATG5 and ATG7. However, ATG9A is not required, suggesting that clockophagy employs selective components of autophagy and does not rely on complete autophagosome biogenesis [18].

The original study defining clockophagy as a selective autophagy process favoring ferroptosis identified the EGLN2/PHD1–HIF-1α axis as a key downstream mechanism [18]. BMAL1/ARNTL is functionally connected with hypoxia signaling: it interacts with HIF-1α, enhances HIF-1α transcriptional activity, and supports HIF-1α protein stability, whereas HIF-1α can feed back onto core clock gene expression [155]. In RSL3-induced ferroptosis, BMAL1/ARNTL degradation increases EGLN2/PHD1 expression [30]. EGLN2/PHD1 then promotes HIF-1α hydroxylation and ubiquitin–proteasomal degradation [156], thereby reducing HIF-1α stability and transcriptional activity. Because HIF-1α regulates lipid-buffering molecules such as FABP3 and FABP7 [157], its reduction weakens lipid storage capacity and enhances lipid peroxidation. This BMAL1–EGLN2–HIF-1α pathway provides a mechanistic basis for how clockophagy promotes ferroptotic cell death in these experimental systems. The proposed molecular mechanism of clockophagy-mediated ferroptotic regulation is summarized in Figure 5.

#### 5.3.2. Clockophagy in Disease-Related Ferroptotic Signaling

In addition to altering lipid-buffering capacity through the EGLN2/PHD1–HIF-1α pathway, clockophagy may also intersect with anti-ferroptotic defense systems. Ferroptosis is restrained by antioxidant pathways that limit lipid peroxide accumulation, particularly the SLC7A11–GSH–GPX4 axis. SLC7A11 supports cystine uptake and glutathione synthesis, whereas GPX4 uses glutathione to detoxify lipid hydroperoxides [140]. Therefore, when BMAL1/ARNTL stability is altered together with SLC7A11 or GPX4 activity, ferroptotic sensitivity may also be affected.

Evidence from non-AD models supports this possibility. In hepatocellular carcinoma models, the DCAF7/USP2 complex promotes BMAL1 deubiquitination and stabilizes BMAL1 protein, thereby reducing its interaction with SQSTM1/p62 and limiting p62-mediated autophagic degradation. Stabilized BMAL1 enhances HIF-1α-related transcriptional activity, upregulates SLC7A11 expression, strengthens antioxidant defense, and suppresses lipid peroxidation and ferroptotic sensitivity [27]. Conversely, in gastric cancer cells, MT1G overexpression has been reported to inhibit GPX4 and promote SQSTM1/p62-associated BMAL1/ARNTL autophagy through the GPX4/SQSTM1 axis. This was accompanied by enhanced autophagic activity, lipid droplet autophagy, mitochondrial abnormalities, reduced antioxidant capacity, increased lipid peroxidation-related stress, and suppression of cancer cell proliferation, migration, and invasion [28]. Together, these findings suggest that BMAL1/ARNTL stability can influence ferroptotic regulation through SLC7A11- and GPX4-related antioxidant defense pathways. Although not a clockophagy study, recent neuronal evidence further shows that GPX4 dysfunction alone can drive ferroptotic neurodegeneration in mouse and patient-derived human neural models [143].

Additional evidence from non-AD models further supports a broader relationship between circadian regulators, autophagy, lipid metabolism, and cell-death signaling. In oral cancer models, BMAL1 overexpression has been reported to induce autophagy-dependent cell death and inhibit cell proliferation, accompanied by reduced expression of the autophagy receptor p62 and the anti-apoptotic protein BCL-2 [158]. In hepatocytes, disruption of rhythmic REV-ERBα oscillation leads to loss of rhythmic expression of autophagy-related proteins, including mTOR, BECN1, LC3, and p62, thereby impairing autophagic flux and promoting lipid droplet accumulation [159]. Although ferroptosis was not directly examined in this hepatocyte study, these findings indicate that circadian disruption can alter autophagic regulation and lipid metabolic homeostasis. In depression-related sleep disturbance models, Semen Ziziphi Spinosae has been reported to modulate BMAL1-dependent clockophagy through the cAMP signaling pathway and improve sleep-related phenotypes [29]. This observation suggests that clockophagy may also be responsive to upstream neuromodulatory pathways involved in sleep regulation. Because tumor and hepatic cells differ substantially from post-mitotic neural cells in proliferative state, metabolism, hypoxia signaling, autophagy dependence, iron handling, and redox regulation, these findings should be regarded as mechanistic proof-of-principle rather than direct evidence for the same pathway in AD.

Taken together, current evidence from non-AD models indicates that clockophagy can regulate ferroptosis via lipid-buffering pathways and anti-ferroptotic defense systems, while broader circadian–autophagy mechanisms may also influence lipid metabolism and cell-death signaling. It remains to be further investigated whether these mechanisms contribute to AD-related neurodegenerative processes. Mechanistic evidence for clockophagy-related ferroptotic regulation from non-AD models is summarized in Figure 6.

### 5.4. Clockophagy as a Potential Link Between Sleep Disturbance and AD

The relevance of clockophagy to AD should be considered in the broader setting of sleep–circadian disruption, BMAL1 dysfunction, oxidative stress, and lipid metabolic imbalance. BMAL1 is not only a core circadian transcription factor but also participates in several AD-related homeostatic processes. BMAL1 deficiency has been reported to disrupt the rhythmic clearance of Aβ in the brain, increase APOE expression, and facilitate amyloid plaque deposition in the hippocampus [19]. Altered BMAL1 rhythmicity has also been associated with lipid metabolic abnormalities, increased lipid peroxidation, and impaired microglial clearance of tau, processes that may contribute to synaptic dysfunction and cognitive impairment [20,23,24]. In addition, in a non-AD neurodegeneration model, BMAL1/Nrf2-related signaling was associated with antioxidant defense, ferroptotic stress, and mitochondrial autophagy [25]. Parallel neuronal evidence also supports the protective role of Nrf2 against ferroptotic stress [56]. Consistent with these observations, BMAL1-deficient mice exhibit circadian disruption, age-related pathologies, and cognitive impairment [21,22].

Sleep disturbance may further intensify this BMAL1-centered vulnerability. OSA, characterized by chronic intermittent hypoxia, has been associated with increased risk of cognitive decline and AD-related pathology [160]. Clinical studies have reported a high prevalence of sleep-related breathing disorders in patients with dementia, with some estimates exceeding 70% [161]. Experimental studies further show that intermittent hypoxia can induce AD-related phenotypes, including memory deficits and impaired synaptic plasticity [162]. Mechanistically, intermittent hypoxia may promote tau seeding and phosphorylation, increase APP expression, and induce mitochondrial dysfunction and neuroinflammation, partly through mTOR-related signaling [163].It can also disrupt microglial metabolism, favor pro-inflammatory microglial activation, compromise blood–brain barrier integrity, and increase ROS production, thereby aggravating synaptic injury and cognitive decline [164,165,166]. In AD, OSA-related intermittent hypoxia may further disrupt circadian organization, while AD-related circadian impairment may in turn destabilize sleep–wake regulation [167]. At the cellular level, hypoxia can disturb lipid homeostasis by expanding endoplasmic reticulum–mitochondria contact sites, inducing mitochondrial ROS generation, and promoting mitochondrial fission, thereby favoring lipid peroxidation and ferroptosis-related stress [168]. Together with BMAL1 dysregulation, hypoxia-associated oxidative stress may increase lipid peroxidation and create conditions that favor ferroptosis-related vulnerability.

Evidence from non-AD experimental models indicates that HIF-1α plays a key role in linking adaptive metabolic responses to hypoxic stress, lipid metabolism, and iron metabolism. It induces various adaptive mechanisms such as metabolic reprogramming, angiogenic activation, lipid accumulation, and regulation of iron metabolism-related proteins, including TfR1 and NCOA4, during hypoxia [169,170]. These responses may help maintain lipid and iron homeostasis by limiting excessive lipid peroxidation and iron-dependent oxidative damage. However, prolonged or repeated hypoxic stress, as may occur during OSA, may compromise this adaptive capacity and contribute to mitochondrial dysfunction, lipid dyshomeostasis, and oxidative stress [171,172,173].

This hypoxic adaptive network is closely connected with circadian regulation. BMAL1-containing clock complexes have been reported to interact with HIF-1α, and BMAL1 can enhance HIF-1α transcriptional activity and support HIF-1α protein stability [155,174]. Conversely, HIF-1α can directly bind to the promoters of core clock genes, thereby feeding back onto circadian clock function, while its own accumulation appears to be time-dependent and influenced by BMAL1 [175].

BMAL1 deficiency increases neuronal oxidative damage and alters the expression of redox defense genes [26]. Intraventricular injection of Aβ aggregates can disrupt the circadian oscillation of protein carbonyl levels, induce phase shifts in clock gene expression, and differentially affect the expression and activity of CAT and GPX4. These findings suggest that Aβ may interfere with the formation of the BMAL1 and CLOCK heterodimer, thereby disrupting the rhythmic transcriptional regulation of antioxidant enzymes and ultimately leading to redox imbalance [31]. BMAL1/Nrf2 signaling has also been implicated in redox regulation in non-AD experimental models [25], while neuronal evidence indicates that NRF2 upregulation can reduce ferroptotic susceptibility [56]. The presence of AD-related factors may interfere with the functioning of this adaptive mechanism. For example, PSEN1 malfunction has been shown to compromise the activation of HIF-1α by insulin, thereby compromising protection against damage in response to oxygen or metabolic stress [176]. Together, these findings suggest that BMAL1 dysfunction may compromise both circadian antioxidant regulation and HIF-1α-dependent hypoxic adaptation.

In this context, clockophagy may represent a potential mechanistic link between sleep disturbance and AD. Evidence from non-AD ferroptosis-related models indicates that SQSTM1/p62-mediated BMAL1/ARNTL degradation can influence HIF-1α signaling, lipid-buffering capacity, antioxidant defense, and ferroptosis-related cell fate. In AD, sleep fragmentation, intermittent hypoxia, impaired circadian entrainment, Aβ-related clock disruption, and BMAL1 dysfunction may affect overlapping redox and lipid regulatory pathways. Recent neuronal evidence further shows that GPX4 dysfunction can drive ferroptotic neurodegeneration with Alzheimer’s-like molecular signatures [143]. However, this finding does not establish a clockophagy–ferroptosis pathway in AD. Whether SQSTM1/p62-mediated BMAL1/ARNTL degradation occurs in AD and whether clockophagy contributes to Aβ pathology, tau dysfunction, synaptic impairment, or cognitive decline remain to be determined. The evidence supporting each component of this framework, together with the major unresolved questions, is summarized in Table 2. The proposed mechanistic framework is illustrated in Figure 7.

## 6. Future Research Directions and Challenges

Both sleep and circadian disturbances are increasingly recognized as integral components of the disease process associated with AD. Evidence summarized in this review indicates a bidirectional relationship in which AD pathology disrupts sleep–wake and circadian organization, whereas persistent sleep and circadian disturbances may further compromise neural resilience under proteotoxic, metabolic, hypoxic, and oxidative stress. BMAL1 occupies a potentially important position at the intersection of circadian transcription, autophagy–lysosome function, redox regulation, and lipid metabolism. In this context, clockophagy, defined as SQSTM1/p62-mediated selective autophagic degradation of BMAL1, provides a plausible mechanistic basis for linking circadian instability with lipid peroxidation and ferroptotic vulnerability. Rather than representing a simple linear cascade, this framework may reflect a network of reciprocal interactions in which circadian disruption, impaired proteostasis, metabolic stress, and lipid-redox imbalance reinforce one another and create conditions that favor Aβ accumulation, tau dysfunction, synaptic impairment, and neuroinflammation.

This framework also has potential therapeutic implications at behavioral, clinical, and molecular levels. Management of specific sleep and circadian phenotypes, including insomnia, sleep-disordered breathing, and circadian disruption, remains the most immediately applicable approach, with strategies such as CBT-I, PAP/CPAP for OSA, sleep hygiene, and regular sleep scheduling. Melatonin-related treatments and orexin receptor antagonists have also been evaluated clinically [177,178]. In experimental AD models, exercise and time-restricted feeding have been reported to improve circadian function and attenuate neuropathological or cognitive abnormalities [179,180]. At the molecular level, strategies targeting BMAL1 homeostasis, autophagy–lysosome function, endogenous antioxidant defenses, lipid peroxidation, and ferroptotic vulnerability remain preclinical. If excessive SQSTM1/p62-dependent BMAL1 degradation is eventually demonstrated in AD, modulation of BMAL1 stability or its selective autophagic turnover could represent an additional therapeutic opportunity. However, such interventions should aim to restore pathway homeostasis rather than globally suppress autophagy, because basal autophagy remains essential for neuronal proteostasis and Aβ/tau clearance. The context-dependent effects of these pathways further suggest that therapeutic efficacy may vary according to treatment timing, disease stage, and cellular state, supporting the development of mechanism-based chronotherapeutic approaches. The long-term effects of sleep- and circadian-directed interventions on AD progression nevertheless remain uncertain.

Several major barriers currently limit translation of this framework. Evidence for clockophagy-dependent ferroptosis has been derived predominantly from experimental systems outside the AD-affected nervous system, whereas the roles of BMAL1 in redox regulation, autophagy–lysosome function, lipid metabolism, and Aβ homeostasis have largely been characterized in separate experimental settings. Current evidence therefore supports mechanistic convergence but does not yet demonstrate that these processes form a unified clockophagy–ferroptosis pathway in AD. This limitation is particularly important when interpreting findings from cancer and other non-neural models, in which proliferative state, metabolic demand, hypoxia signaling, autophagy dependence, iron handling, and redox regulation may differ substantially from those of neurons and glial cells. Such studies provide valuable mechanistic proof-of-principle, but their relevance to AD requires validation in disease-relevant neural systems. In addition, most experimental models do not fully reproduce the chronic progression, cellular heterogeneity, aging-related changes, and circadian variability of human AD. The lack of dynamic biomarkers reflecting BMAL1 turnover, autophagic flux, and lipid-redox stress further complicates mechanistic classification and clinical translation.

A central challenge will therefore be to determine whether altered BMAL1 abundance in AD reflects changes in circadian transcription and rhythmicity, altered proteasomal turnover, or a shift toward selective lysosomal degradation. Resolving these possibilities will require dynamic assessment of BMAL1 protein stability together with the pathways that regulate its turnover. Time-resolved analysis of BMAL1/ARNTL in relation to SQSTM1/p62, relevant autophagy machinery, and lysosomal activity may help establish whether BMAL1 is selectively recruited to autophagic structures under AD-related stress conditions. Complementary biochemical and imaging approaches could further clarify the spatial and molecular interaction of BMAL1/ARNTL with SQSTM1/p62 and lysosomal compartments. Importantly, these changes should be examined together with downstream alterations in HIF-1α signaling, antioxidant defense, lipid peroxide accumulation, and ferroptotic susceptibility. Studies across AD-relevant neuronal and glial models, human iPSC-derived systems, animal models, and human tissue will be required to determine whether clockophagy represents a disease-associated mechanism in AD or a context-dependent cellular stress response.

Future studies should also account explicitly for cell type and circadian phase when interpreting BMAL1-related changes. BMAL1-dependent regulation of autophagy, lysosomal function, lipid metabolism, and redox homeostasis may differ among neurons, astrocytes, microglia, and other neural and vascular cell populations. Moreover, a reduction in BMAL1 abundance at a single time point cannot distinguish reduced circadian amplitude, phase shifting, loss of rhythmicity, or true pathological protein degradation. Time-series and circadian-phase-resolved measurements in defined cell populations will therefore be essential for distinguishing altered rhythmic regulation from abnormal protein turnover. More broadly, future studies should move from broad associations toward precise, temporally resolved characterization of individual sleep–circadian phenotypes and their underlying molecular states. Development of biomarkers that simultaneously reflect sleep–circadian status, BMAL1 homeostasis, autophagic activity, and lipid-redox stress will be particularly important. Objective, repeated, and standardized sleep–circadian phenotyping may provide a methodological basis for cross-cohort comparisons, longitudinal studies, and intervention trials [181]. In parallel, integration of sleep medicine, circadian biology, neurodegeneration research, lipidomics, spatial and single-cell approaches, and computational modeling may help resolve the temporal and cellular heterogeneity of this regulatory network. Ultimately, these advances will determine whether clockophagy represents a causal component of AD pathogenesis or a context-dependent modifier of ferroptotic vulnerability, and whether this framework can be translated into biomarker-guided and temporally informed therapeutic strategies.

## 7. Conclusions

Sleep disturbances and circadian dysfunction are increasingly recognized as important components of AD and are closely associated with oxidative stress, lipid metabolic imbalance, autophagy–lysosome dysfunction, and ferroptotic vulnerability. This review systematically summarizes the relationships among sleep–circadian disruption, AD-related pathology, BMAL1 homeostasis, autophagic regulation, lipid peroxidation, and ferroptosis. Particular attention is given to clockophagy, an SQSTM1/p62-mediated selective autophagic process that promotes BMAL1/ARNTL degradation and ferroptosis in established experimental models. By integrating these lines of evidence, we propose that clockophagy may represent a potential molecular interface linking sleep–circadian dysfunction with altered lipid-redox homeostasis and ferroptotic vulnerability in AD.

However, direct evidence demonstrating bona fide clockophagy in AD neurons, glia, or animal models is currently lacking. Thus, the proposed sleep–circadian–clockophagy–ferroptosis axis should be regarded as a testable mechanistic framework rather than an established pathway in AD pathogenesis. Further clarification of this relationship may deepen our understanding of how circadian and sleep disturbances interact with cellular stress responses in AD and provide a conceptual basis for mechanism-oriented and temporally informed therapeutic strategies.

## Figures and Tables

**Figure 1:**
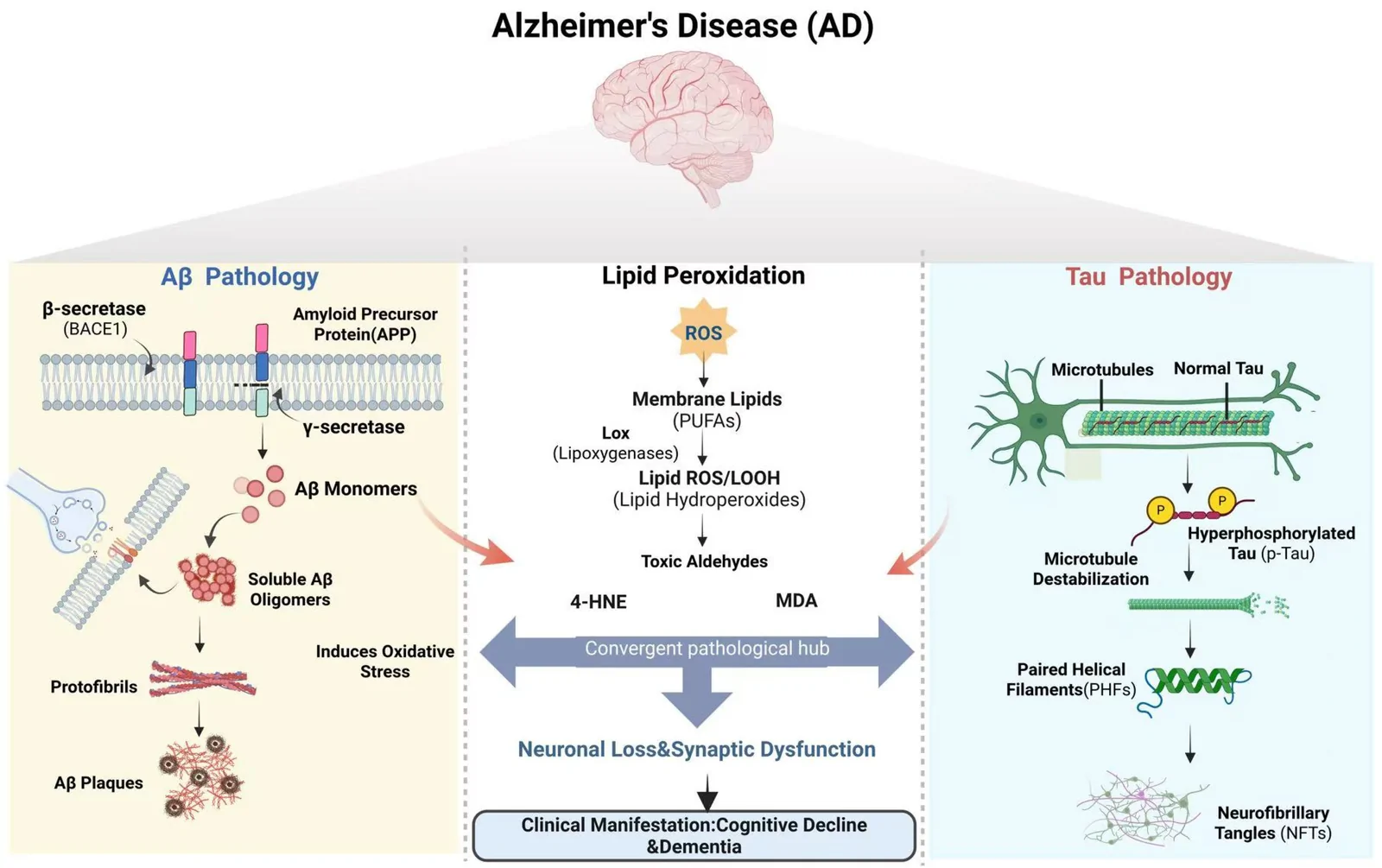
**Proposed role of lipid peroxidation as a convergent pathological hub in AD.** The schematic illustrates proposed interactions among Aβ pathology, tau abnormalities, and lipid oxidative stress in AD. Aβ aggregation and its interactions with neuronal membranes and redox-active metals may enhance ROS generation and lipid oxidation. Tau abnormalities may interact with altered lipid metabolism and oxidative stress, whereas lipid peroxidation generates lipid hydroperoxides and reactive aldehydes such as 4-HNE and MDA. These processes can impair membrane integrity, organelle function, protein homeostasis, and synaptic function and may thereby increase neuronal vulnerability. Created with BioRender.com. Jianqin Mao. (2026) https://app.biorender.com/illustrations/693546458da1c5bdac6b2b47.

**Figure 2:**
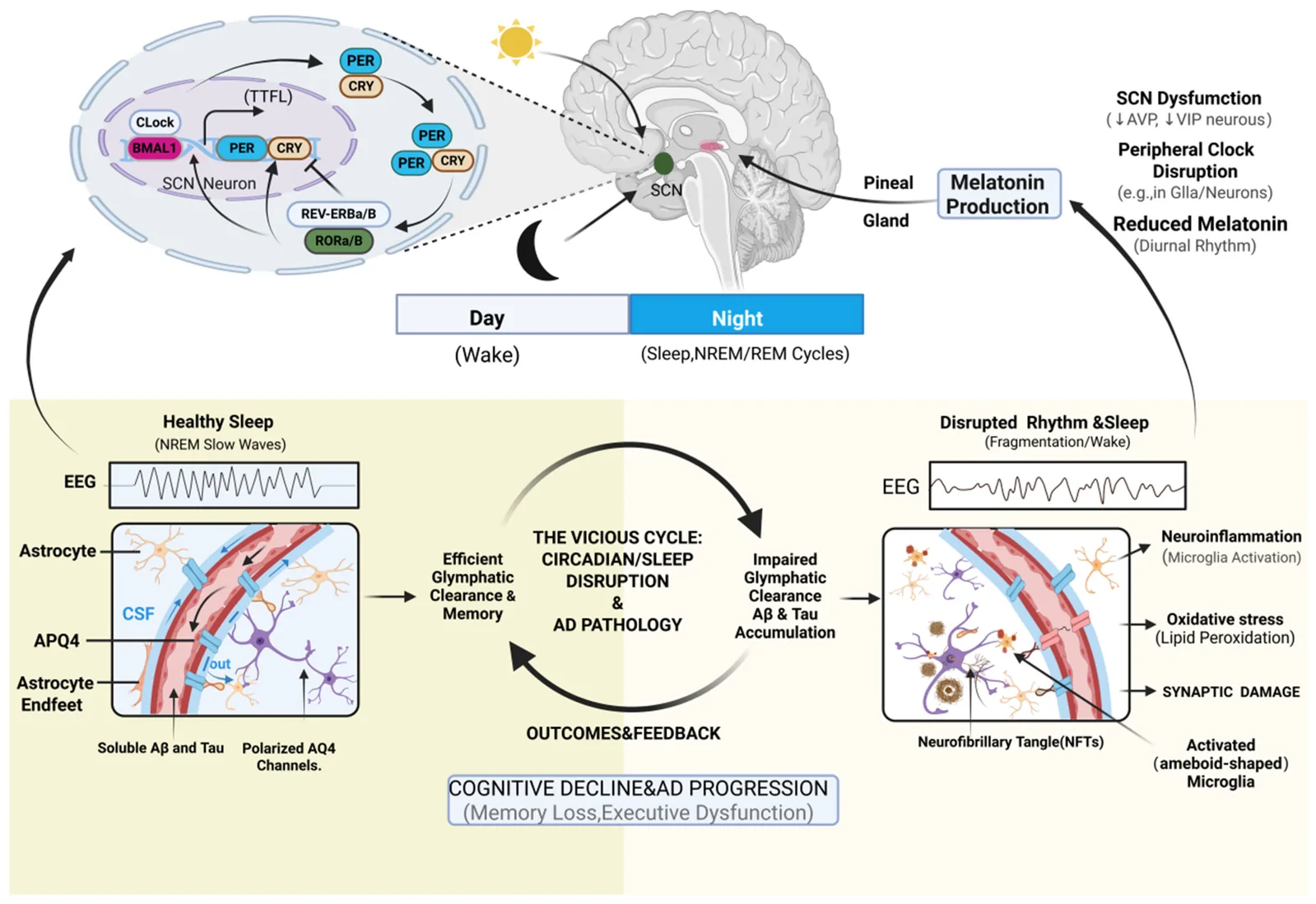
**Circadian and sleep disruption may contribute to AD through converging pathological mechanisms.** The SCN coordinates circadian timing through molecular clock components, including CLOCK, BMAL1, PER, and CRY, and contributes to the temporal organization of sleep–wake behavior, hormonal secretion, arousal, feeding, and cognitive processes. Sleep is additionally regulated by homeostatic mechanisms that are distinct from circadian timing. Experimental evidence, predominantly from animal studies, suggests that NREM/slow-wave sleep may facilitate CSF and glymphatic transport and thereby influence the handling of soluble Aβ, tau-related species, and other metabolites. In AD, circadian misalignment and specific sleep disturbances may interact with impaired proteostasis, altered synaptic plasticity, neuroinflammation, oxidative stress, and lipid metabolic dysfunction. Created with BioRender.com. Jianqin Mao. (2026) https://app.biorender.com/illustrations/694ff24fcb40d7da24e422f0.

**Figure 3:**
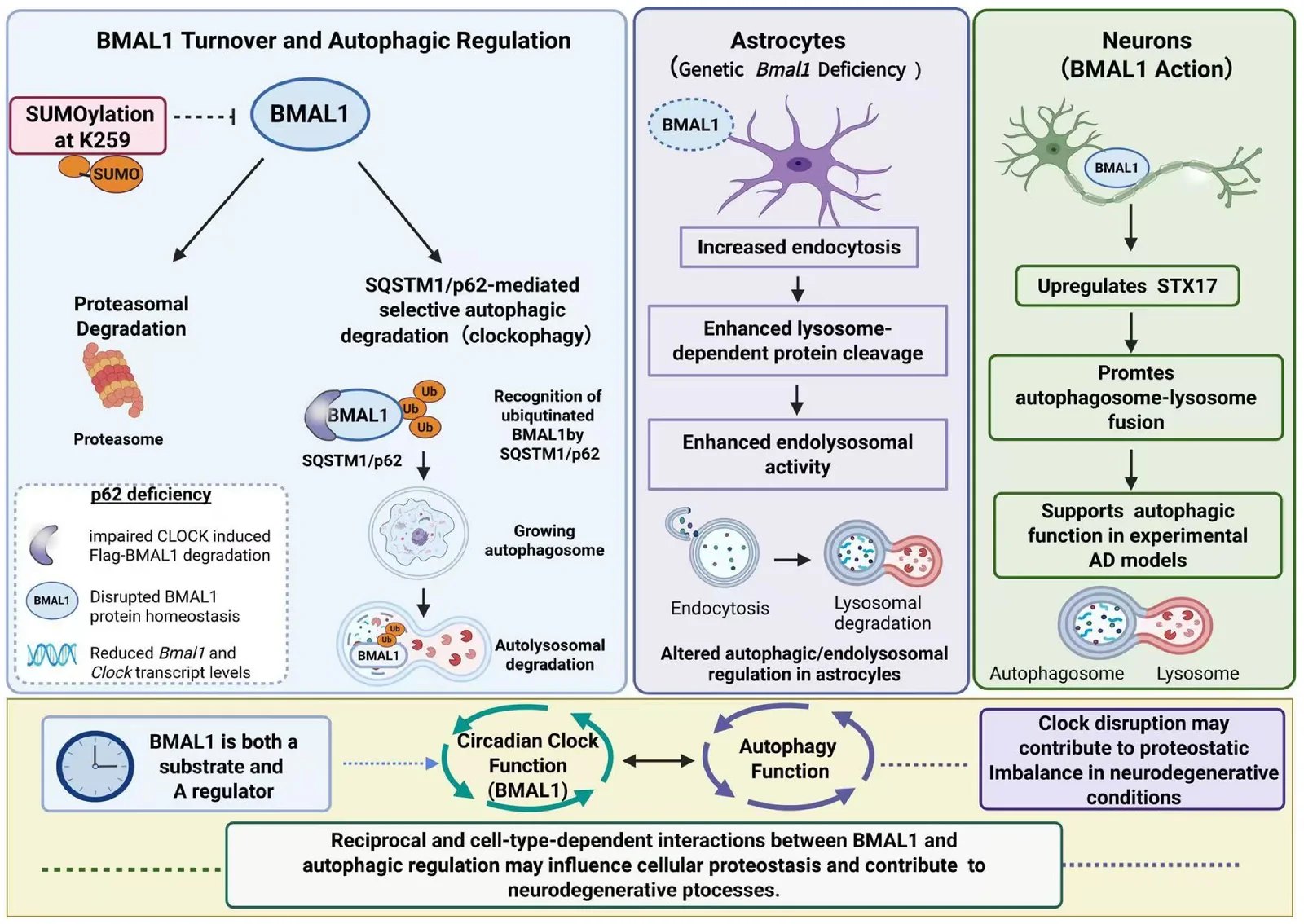
**BMAL1-centered autophagic regulation.** BMAL1/ARNTL protein stability is regulated through proteasomal and autophagic degradation pathways, while SUMOylation at K259 contributes to the regulation of BMAL1 stability and circadian transcriptional activity. In clockophagy, SQSTM1/p62 functions as a selective autophagy receptor that promotes BMAL1/ARNTL degradation. Beyond being an autophagic substrate, BMAL1 also regulates autophagic processes in neural cells. In astrocytes, genetic BMAL1 deficiency is associated with enhanced endolysosomal activity, whereas in hippocampal neurons BMAL1 promotes STX17-dependent autophagosome–lysosome fusion. Together, these findings highlight the reciprocal relationship between BMAL1 stability and autophagic regulation and support further investigation of the potential relevance of this axis to neurodegenerative processes. Created with BioRender.com. Jianqin Mao. (2026) https://app.biorender.com/illustrations/6a4ddaa54a426a7a6eccf968.

**Figure 4:**
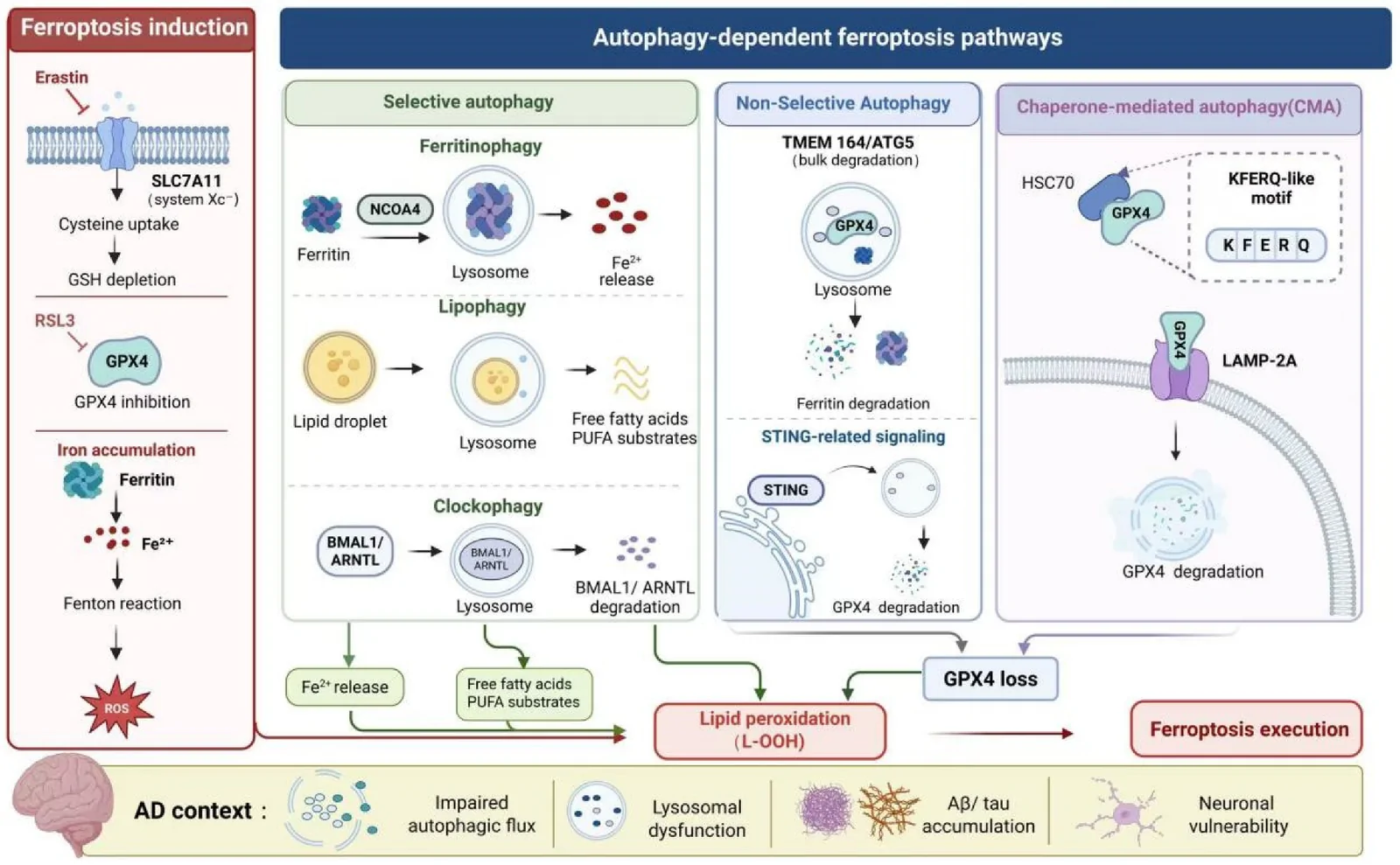
**Autophagy, lipid peroxidation, and ferroptotic regulation in AD.** Autophagy may regulate ferroptotic susceptibility through selective, non-selective, and chaperone-mediated pathways. Selective autophagy includes ferritinophagy, lipophagy, and clockophagy, which may affect ferroptotic vulnerability by regulating iron storage, lipid droplet turnover, and BMAL1/ARNTL stability, respectively. Non-selective autophagy may influence ferroptotic susceptibility through TMEM164/ATG5-dependent bulk degradation of GPX4 and ferritin, whereas STING-related autophagic signaling has also been implicated in GPX4 degradation. CMA facilitates GPX4 degradation through HSC70-mediated recognition of its KFERQ-like motif and LAMP2A-dependent lysosomal delivery. Importantly, the effects of autophagy on ferroptosis are context-dependent and may be either pro-ferroptotic or protective. These mechanisms provide a framework for understanding how autophagic dysregulation may intersect with lipid peroxidation and ferroptotic susceptibility in AD. Created with BioRender.com. Jianqin Mao. (2026) https://app.biorender.com/illustrations/696bb96304d8cc8b4095b8dc.

**Figure 5:**
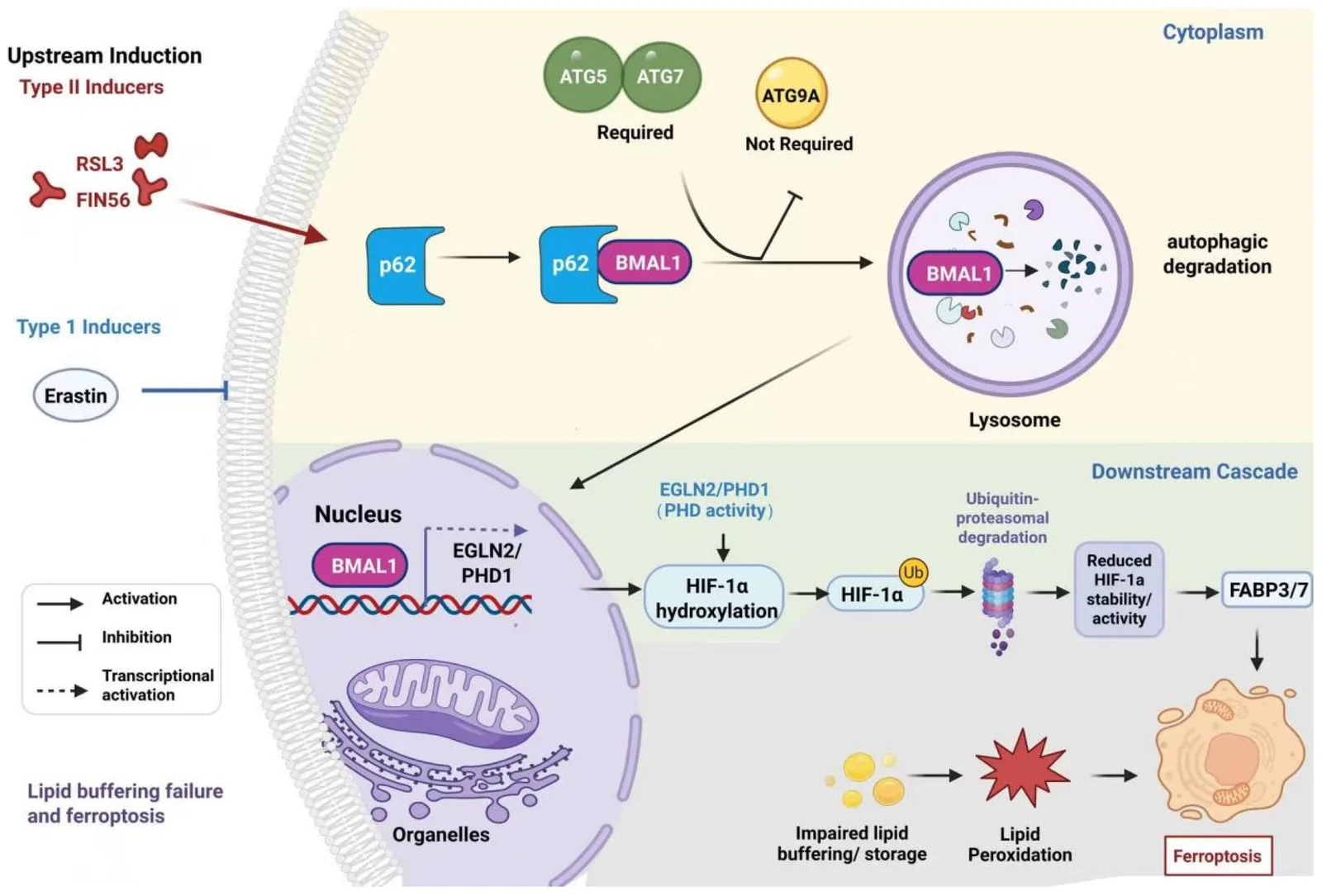
**Molecular mechanisms underlying clockophagy-mediated ferroptotic regulation.** Type II ferroptosis inducers, including RSL3 and FIN56, promote SQSTM1/p62-mediated selective autophagic degradation of the core circadian regulator BMAL1/ARNTL. This process requires autophagy-related proteins ATG5 and ATG7. BMAL1/ARNTL degradation is associated with increased EGLN2/PHD1 expression, which promotes HIF-1α hydroxylation and ubiquitin–proteasomal degradation, resulting in reduced HIF-1α stability and transcriptional activity. The subsequent reduction in FABP3/7 expression impairs lipid buffering capacity, enhances lipid peroxidation, and promotes ferroptotic cell death. Created with BioRender.com. Jianqin Mao. (2026) https://app.biorender.com/illustrations/6a5dd843f1d87e91d3277389.

**Figure 6:**
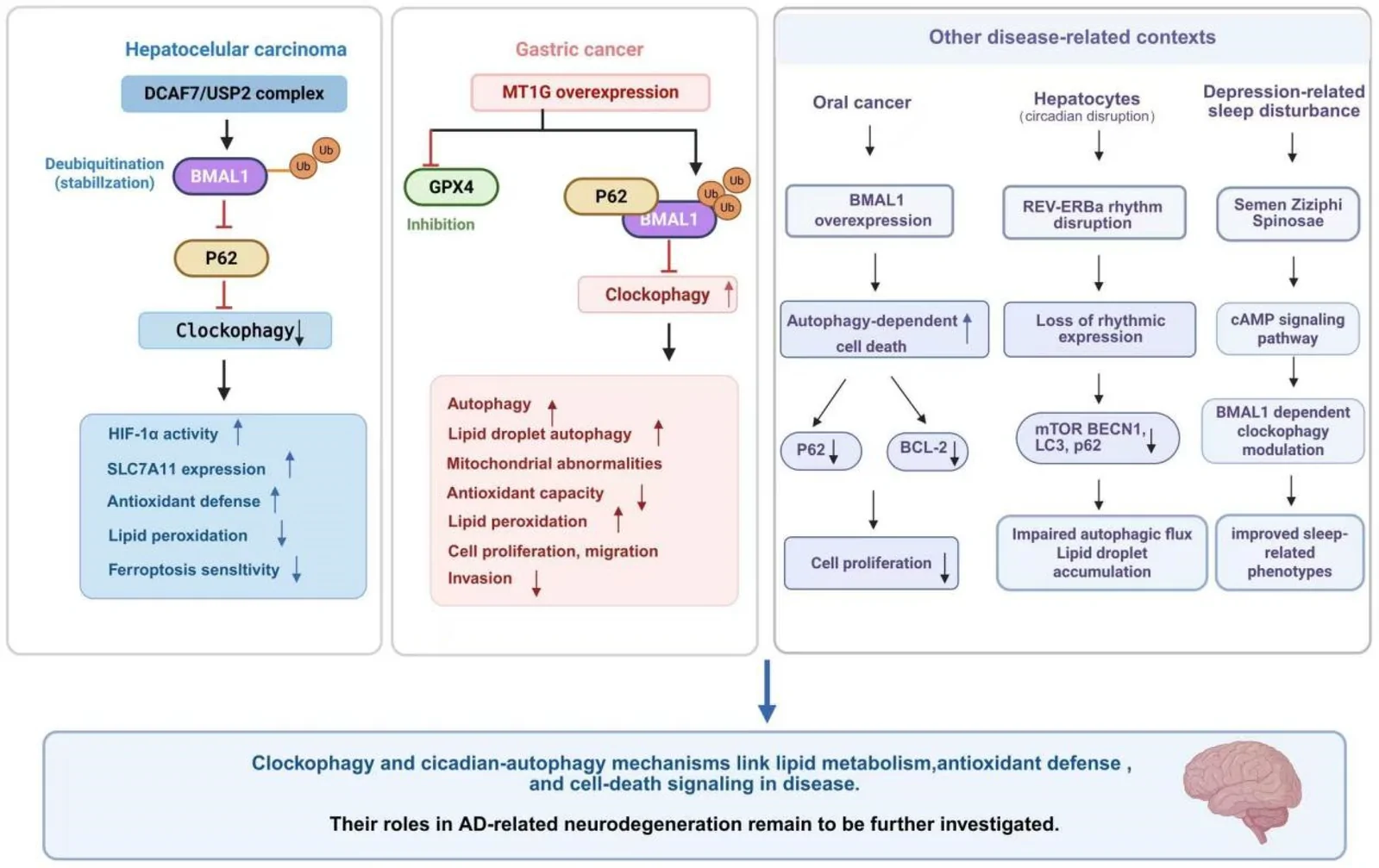
**Clockophagy and ferroptotic regulation: mechanistic evidence from non-AD models.** Clockophagy-associated pathways regulate ferroptotic signaling through modulation of circadian protein stability, autophagy, antioxidant defense, and lipid metabolism. In hepatocellular carcinoma and gastric cancer models, alterations in BMAL1/ARNTL stability are associated with changes in SQSTM1/p62-mediated autophagy, SLC7A11/GPX4-dependent antioxidant defense, lipid peroxidation, and ferroptotic responses. Additional non-AD models indicate that circadian regulators can influence autophagic flux and lipid metabolic homeostasis through BMAL1-dependent mechanisms and broader circadian–autophagy regulation. These findings provide mechanistic evidence from non-AD systems. Created with BioRender.com. Jianqin Mao. (2026) https://app.biorender.com/illustrations/6a4dd9038bc5f36134c89424.

**Figure 7:**
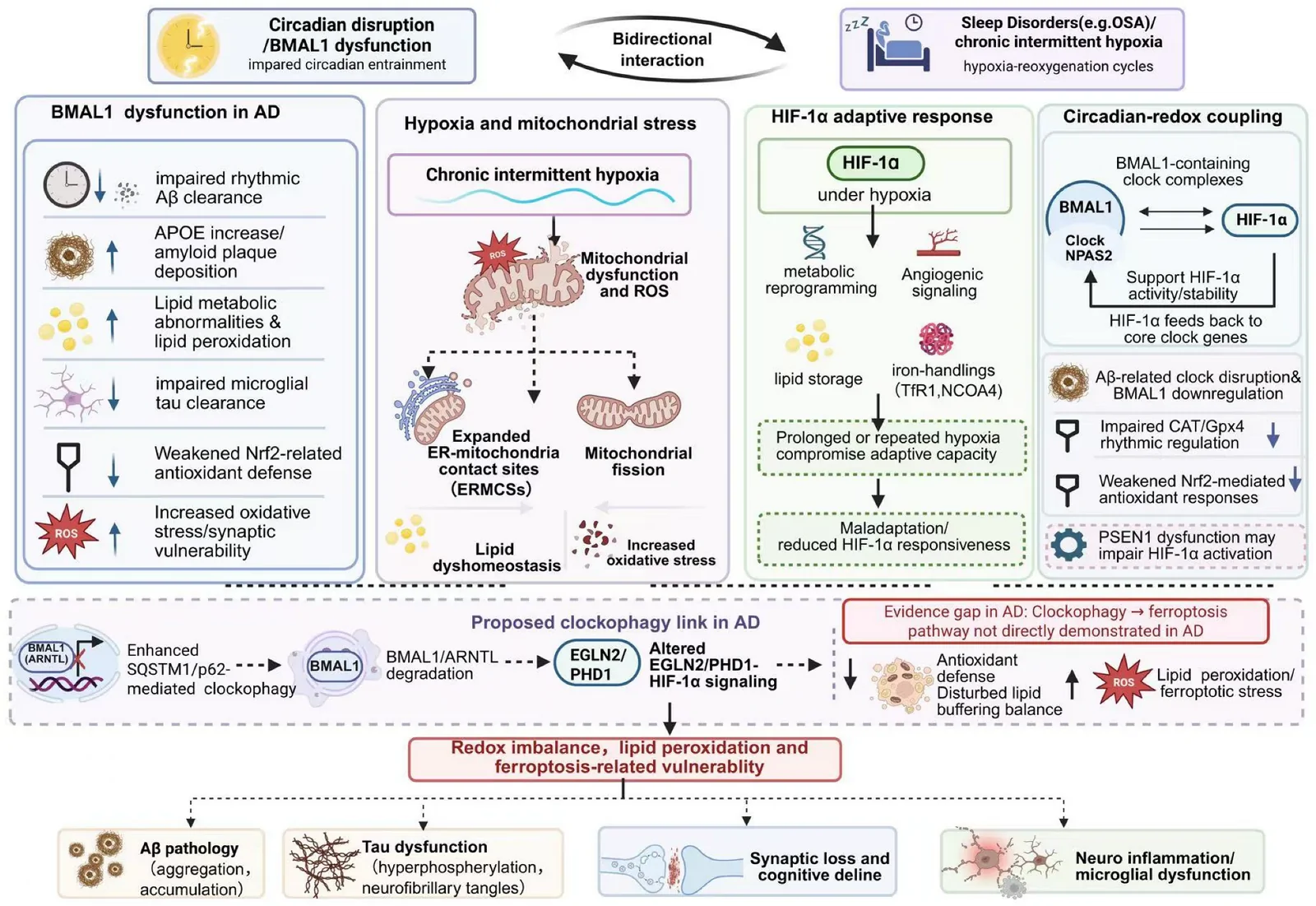
**Proposed role of clockophagy in linking sleep–circadian disruption to ferroptotic vulnerability in AD.** Sleep-disorder-associated hypoxia and BMAL1 dysregulation may converge to promote circadian misalignment, oxidative stress, and lipid dyshomeostasis. Enhanced SQSTM1/p62-mediated clockophagy promotes BMAL1 degradation, and reduced BMAL1 may alter EGLN2–HIF-1α signaling, impairing metabolic adaptation and increasing susceptibility to lipid peroxidation. Concurrent hypoxia may induce mitochondrial dysfunction, ROS accumulation, expansion of ER–mitochondria contact sites, and ferroptosis-related stress. BMAL1 is also linked to Nrf2 and GPX4 related antioxidant defense pathways. These interacting mechanisms may contribute to conditions favoring Aβ/tau pathology, neuroinflammation, and synaptic dysfunction in AD. In the schematic, solid arrows denote experimentally supported relationships, dashed arrows indicate mechanistic evidence derived mainly from non-AD models, and dotted arrows indicate proposed AD-specific links. The clockophagy–ferroptosis pathway has not been directly demonstrated in AD. Created with BioRender.com. Yudan Zhang. (2026) https://app.biorender.com/illustrations/695e6dc1068812b2cd10adb6.

**Table 1:** Evidence hierarchy supporting a proposed clockophagy–ferroptosis framework in AD.

Mechanistic Link	AD-Specific Evidence	Supporting Evidence from Non-AD Models	Current Evidentiary Status	Key Unresolved Question	Representative Evidence
Sleep/circadian disruption ↔ AD pathology	Substantial clinical and experimental evidence	Substantial	Supported bidirectional association	Temporal sequence and disease-stage dependence	[5,7,9,10,11,12,13,14]
BMAL1 dysfunction → AD-related pathology	Moderate experimental evidence	Substantial	Supported, context dependent	Cell-type and circadian-phase specificity	[19,20,21,22]
BMAL1 dysregulation → redox and lipid homeostasis impairment	Limited-to-moderate	Substantial	Mechanistically supported	Contribution to ferroptotic vulnerability in AD	[20,23,24,25,26]
Clockophagy → ferroptosis	None demonstrated in AD	Direct mechanistic evidence	Established outside AD	Demonstrate SQSTM1-dependent BMAL1 degradation in AD	[18,27,28]
Sleep/circadian disturbance → Clockophagy	None demonstrated in AD	Limited/indirect	Hypothesis in AD	Identify physiological/pathological triggers	[29]
Clockophagy → ferroptotic vulnerability in AD	No direct evidence	Mechanistic foundation established outside AD	Central hypothesis	Establish causal pathway in AD models	[18,19,27,28,30,31]
Modulating Clockophagy → AD protection	No direct evidence	Limited/indirect	Testable prediction	Establish efficacy without disrupting basal autophagy/circadian function	[27,29,32]

**Table 2:** Mechanistic interplay among AD, sleep disturbance, and circadian dysfunction.

Category	Alzheimer’s Disease	Sleep Disturbance	Circadian Disruption	Autophagy	Ferroptosis	Clockophagy
**Definition**	Neurodegenerative disorder with Aβ and tau aggregation	Altered sleep duration, continuity, architecture, or breathing	Altered circadian phase, amplitude, or entrainment	Lysosome-dependent degradation maintaining cellular homeostasis	Iron-dependent lipid peroxidation driven cell death	SQSTM1/p62-mediated degradation of BMAL1
**Key features**	Memory loss, cognitive decline, synaptic dysfunction	Insomnia; fragmentation; reduced slow-wave sleep; OSA	Irregular sleep–wake timing; reduced rhythm amplitude; melatonin disruption	Altered autophagic flux; lysosomal dysfunction; p62 accumulation	Lipid ROS; GPX4 impairment; iron dyshomeostasis	BMAL1/ARNTL degradation; altered HIF-1α signaling; ferroptotic vulnerability
**Mechanistic links**	Aβ/tau pathology ↔ oxidative and autophagic stress	Sleep loss → impaired clearance and oxidative stress; OSA → intermittent hypoxia	BMAL1 dysregulation → redox and metabolic imbalance	Impaired clearance → protein aggregation; selective autophagy may regulate ferroptosis	Lipid peroxidation → neuronal vulnerability	Proposed link between circadian disruption, BMAL1 dysregulation, and ferroptotic vulnerability
**Associated factors**	Aging; APOE ε4; vascular and metabolic factors	OSA, stress, circadian misalignment	Aging; shift work; irregular light or feeding	ROS; inflammation; mitochondrial damage	Iron overload; PUFA accumulation; antioxidant failure	BMAL1 instability; p62 activation; oxidative or hypoxic stress
**Therapeutic strategies**	Risk reduction; sleep and circadian management	CBT-I; PAP/CPAP; regular sleep scheduling	Light therapy; timed activity or feeding; melatonin	Autophagy or lysosome modulation (preclinical)	Ferroptosis inhibitors; Nrf2/GPX4 modulation(preclinical)	BMAL1 stabilization, p62 modulation or rhythm restoration (hypothetical/preclinical)

## Data Availability

No new data were created or analyzed in this study. Data sharing is not applicable to this article.

## Abbreviations

AD: Alzheimer’s disease; Aβ: Amyloid-beta; EEG: Electroencephalography; EMG: Electromyogram; NREM: Non-rapid eye movement; REM: Rapid eye movement; OSA: Obstructive sleep apnea; NFTs: Neurofibrillary tangles; ROS: Reactive oxygen species; PUFAs: Polyunsaturated fatty acids; AA: Arachidonic acid; AdA: Adrenic acid; MDA: Malondialdehyde; 4-HNE: 4-hydroxynonenal; LPOs: Lipid peroxidation products; SCN: Suprachiasmatic nucleus; PVN: Paraventricular nucleus; DMH: Dorsomedial hypothalamus; SPVZ: Subparaventricular zone; LC: Locus coeruleus; LTP: Long-term potentiation; VIP: Vasoactive intestinal peptide; AVP: Arginine vasopressin; CSF: Cerebrospinal fluid.
